# Cytotoxicity and Apoptotic Effects of Polyphenols from Sugar Beet Molasses on Colon Carcinoma Cells in Vitro

**DOI:** 10.3390/ijms17070993

**Published:** 2016-06-23

**Authors:** Mingshun Chen, Zhengang Zhao, Shujuan Yu

**Affiliations:** 1 College of Light Industry and Food Sciences, South China University of Technology, Guangzhou 510640, China; chenshun1221@163.com; 2 Guangdong Province Key Laboratory for Green Processing of Natural Products and Product Safety, South China University of Technology, Guangzhou 510640, China

**Keywords:** sugar beet molasses, polyphenols, cytotoxicity, Caco-2, apoptosis

## Abstract

Three polyphenols were isolated and purified from sugar beet molasses by ultrasonic-aid extraction and various chromatographic techniques, and their structures were elucidated by spectral analysis. Cytotoxicity and the molecular mechanism were measured by methyl thiazolyl tetrazolium (MTT) assay, flow cytometry, caspase-3 activity assay and Western blot assay. The results showed that gallic acid, cyanidin-3-*O*-glucoside chloride and epicatechin have cytotoxicity to the human colon, hepatocellular and breast cancer cells. Cyanidin-3-*O*-glucoside chloride showed its cytotoxicity against various tumor cell lines, particularly against colon cancer Caco-2 cells with half maximal inhibitory concentration (IC_50_) value of 23.21 ± 0.14 μg/mL in vitro. Cyanidin-3-*O*-glucoside chloride may be a potential candidate for the treatment of colon cancer. In the mechanism study, cyanidin-3-*O*-glucoside chloride increased the ratio of cell cycle at G_0_/G_1_ phase and reduced cyclin D1 expression on Caco-2 cells. Cyanidin-3-*O*-glucoside chloride decreased mutant p21 expression, and increased the ratio of Bax/Bcl-2 and the activation of caspase-3 to induce apoptosis.

## 1. Introduction

Cancer is a world-wide concern that remains a major threat to people’s health, for which current therapeutic approaches are still very limited [1,2]. In the metastatic setting, chemotherapy is the gold standard of palliative therapy [3]. During the past decades, many natural products and derivatives have served as an important source of potent antitumor drugs [4]. Polyphenols have been reported to exhibit several biological activities including antitumor activity [5].

Sugar beet is an important crop for the production of sugar [6,7,8]. Sugar beet molasses (SBM), with a production of more than four million tons per year, is one of the most abundant by-products in sugar beet processing [9]. Sugar beet molasses contain a high content of polyphenols [10,11,12]. Early researchers have evaluated the antioxidant [10,13], anti-inflammatory [14] and DNA-damage-protecting [15] activities of sugar beet molasses, which showed positive results. In our previous study, it was found that ethyl acetate fraction, *n*-butanol fraction and aqueous fraction of SBM extract could inhibit the growth of human hepatocellular HepG2, breast MCF-7, and especially human colon Caco-2 carcinoma cells [6]. However, there has not been any report available on which polyphenols from SBM extract are the active components in the whole process. Moreover, little is known about the cytotoxicity of polyphenols on human colon cancer and their mechanism of action.

In our study, the cytotoxicity effect of three polyphenols of SBM extract against three cancer cell lines was screened. Furthermore, the inhibition to tumor growth in vitro was evaluated with the cyanidin-3-*O*-glucoside chloride (CGC). We also investigated the mechanisms of CGC that inhibited the cell proliferation including apoptosis and cell cycle arrest.

## 2. Materials and Methods

### 2.1. Plant Material

**Figure 1:**
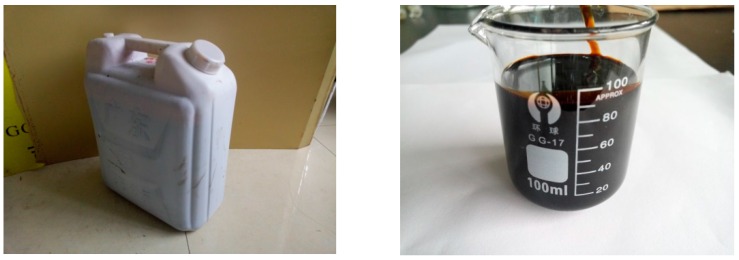
The sugar beet molasses (SBM) of Xinjiang Green Xiang Sugar Industry Co., Ltd., Tacheng, China.

Sugar beet molasses (Figure 1) was provided by Xinjiang Green Xiang Sugar Industry Co., Ltd. (Tacheng, China).

### 2.2. Extraction and Isolation of SBM

The different extracts of SBM were prepared according to our previously described methods [6]. Briefly, 2.0 g sugar beet molasses was extracted by 60 mL 60% (*w*/*v*) ethanol using an RK102H ultrasonic (Bandelin SONOREX, Berlin, Germany). Extraction conditions were ultrasonic power, 450 W, HCl concentration 1.6 mol/L, temperature 40 °C and time 60 min. After centrifugation at 1000× *g* for 10 min, the supernatant was concentrated at 45 °C in vacuum to obtain the total extracted fraction. Then, the total extracted fraction was suspended in distilled water. The resulting solution was successively partitioned with different solvents, which yielded petroleum ether, chloroform, ethyl acetate, *n*-butanol and aqueous fractions. The ethyl acetate fraction was concentrated at 45 °C in vacuum and analyzed.

**Figure 2:**
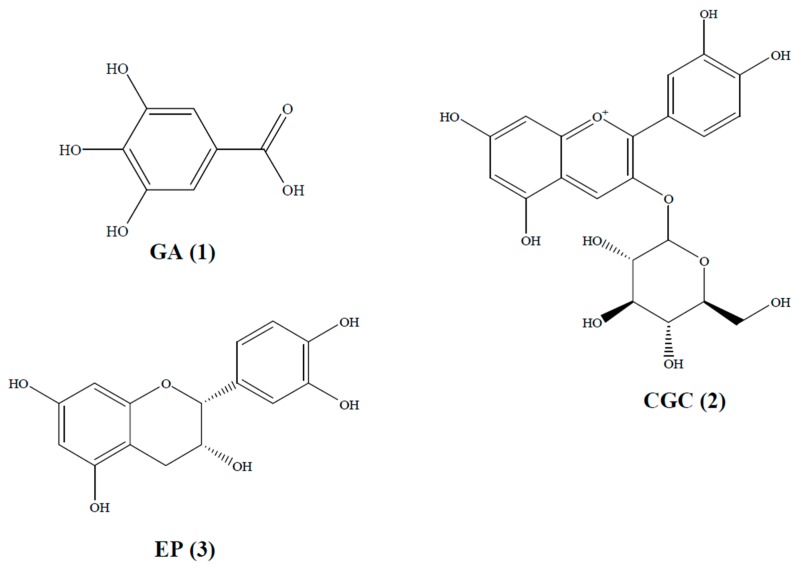
The molecular structures of three SBM extracts. GA: gallic acid, CGC: cyanidin-3-*O*-glucoside chloride, EP: epicatechin.

From the previous research, we could conclude that the ethyl acetate fraction had the most polyphenols [6,7]. The ethyl acetate fraction, which mainly contained polyphenols, showed the highest cytotoxicity in vitro among the five fractions. Thus, the ethyl acetate fraction was subjected to further purification. To put it simply, the ethyl acetate fraction was dissolved and chromatographed over a D101 macroporous resin column (Zhejiang Zhengguang Industrial Co., Ltd., Hangzhou, China), which was eluted with distilled water and 30%, 50%, 70%, and 95% ethanol at a flow rate of 10 mL/min to yield five subfractions. After high-performance liquid chromatography (HPLC) analyses, the 30% ethanol sub-fraction was further purified using a Sephadex LH-20 column chromatography (Shanghai Baoman Biological Technology Co., Ltd., Shanghai, China) and eluted with methanol, followed by a semipreparative HPLC eluting with methanol/water, to yield gallic acid (GA, compound 1), CGC (compound 2) and epicatechin (EP, compound 3). In addition, all spectroscopic data were in complete agreement with the reported data. The structures of the three compounds are shown in Figure 2.

### 2.3. Cell Lines and Cell Culture

Human colon (Caco-2), hepatocellular (HepG2) and breast (MCF-7) cancer cell lines were obtained from the Medical College of Sun Yat-Sen University (Guangzhou, China). The cells were cultured in Dulbecco Modified Eagle Medium (DMEM) containing 10% fetal bovine serum (FBS), 100 units/mL penicillin and 100 μg/mL streptomycin in a humidified incubator with 5% CO_2_ at 37 °C.

### 2.4. Cytotoxicity Assay

The cytotoxicity of the SBM extract against the tumor cells was evaluated using the methyl thiazolyl tetrazolium (MTT) assay [16]. Briefly, cells were harvested during the logarithmic growth phase, seeded at a density of 5 × 10^4^ cells/well onto a 96-well plate and incubated at 37 °C in an atmosphere of 5% CO_2_. After plating overnight, 100 µL serial concentrations of test samples were added to each well. The cells were then incubated at 37 °C for 72 h. Then, 20 μL of MTT solution (5 mg/mL) was added to each well. The absorbance was measured at 490 nm with an ELISA reader (Bio-Rad 680, Hercules, CA, USA). The buffer and 5-fluorouracil (5-FU) were used as the blank and positive control, respectively. The inhibition rate was calculated using the following equation:

(1) $$A=(1-\frac{A_{s}-A_{b}}{A_{c}-A_{b}})\times 100\%$$

where *A* is the cancer cell growth inhibition rate; *A*_c_ is the absorbance of the control; *A*_s_ is the absorbance of sample; and *A*_b_ is the absorbance of the blank. The concentration of the extracts that caused 50% growth inhibition was the half maximal inhibitory concentration (IC_50_) value. The cytotoxicity of the SBM extract was investigated comparatively based on IC_50_ values.

### 2.5. Cell Cycle Analysis by Flow Cytometry

Caco-2 cells were seeded in six-well plates (1 × 10^6^ cells/well). After 24 h plating and then 24 h serum starvation, cells were incubated with the CGC for 4 h with various doses. After that, the cells were harvested by trypsinization, washed thrice with cold phosphate-buffered saline (PBS) and fixed with cold 70% ethanol at 4 °C for 30 min. The cell pellet was incubated with 1.0 mL of PBS containing 100 μg propidium iodide (PI), 100 μg RNase A, and 0.1% Triton X-100 (Shanghai Baoman Biological Technology Co., Ltd., Shanghai, China) at room temperature in the dark for 30 min. The cells were analyzed by flow cytometry (BD, FACSAria II, San Jose, CA, USA).

### 2.6. Cell Apoptosis Analysis by Flow Cytometry

In brief, we treated Caco-2 cells with various doses of CGC for 4 h. Then, cells were harvested, washed and incubated with a solution of Annexin V-fluorescein isothiocyanate (FITC) (Shanghai Baoman Biological Technology Co., Ltd., Shanghai, China) for 20 min and then PI (50 μg/mL) for 10 min. All staining operations were carried out on ice and in the dark. The cells were analyzed by flow cytometry.

### 2.7. Caspase-3 Activity Assay

Caco-2 cells were plated in 96-well plate (1 × 10^6^ cells/well) were treated with 0 to 50 μg/mL of CGC according to the methods in Section 2.5. Then, the treated cells were harvested and lysed by addition of lysis buffer (Thermo Fisher Scientific, Shanghai, China). The samples of the cell lysates were mixed with 100 µM of colorimetric substrate (Ac-DEVD-pNA, Shanghai Baoman Biological Technology Co., Ltd., Shanghai, China) and incubated at 37 °C for 1 h. Alternative activity of the caspase-3 enzyme was described as the cleavage of colorimetric substrate by measuring the absorbance at 405 nm.

### 2.8. Western Blot Assay

Caco-2 cells were treated with various concentrations of CGC for 24 h. Cell lysates were prepared in a buffer containing 0.2% (*w*/*v*) sodium dodecyl sulfate (SDS), 0.5% (*v*/*v*) Triton X-100, 0.5% (*w*/*v*) sodium deoxycholate, 1 mM phenylmethylsulfonyl fluoride (PMSF), and 1% protease inhibitor cocktail. Protein concentration was determined using bicinchoninic acid (BCA) reagent (Pierce Chemical Co., Rockford, IL, USA). The equal amount of protein was electrophoresed on 10% sodium dodecyl sulfate-polyacrylamide gel electrophoresis (SDS-PAGE) and transferred to nitrocellulose membranes. The membranes were blocked with PBS containing 5% low-fat milk for 1 h, incubated overnight with primary antibodies at 4 °C and subsequently incubated with secondary horseradish peroxidase-conjugated, goat anti-rabbit or goat anti-mouse IgG (Abcam, Cambridge, UK). The following antibodies were used: cyclin D1, p21, Bcl-2, Bax and GAPDH from Santa Cruz Biotechnology (Dallas, TX, USA). The blots were visualized using enhanced chemiluminescence (ECL) detection reagents (Amersham, Piscataway, NJ, USA).

### 2.9. Statistical Analysis

All experiments were carried out in triplicate. Means and standard deviations of the data were calculated for each treatment. Analysis of variance (ANOVA) was carried out to determine any significant differences (*p* < 0.05). SPSS software package (SPSS 10.0 for Windows, IBM, New York, NY, USA) was used for statistical calculations.

## 3. Results and Discussion

### 3.1. Evaluation of Cytotoxicity against Tumor Cells

**Figure 3:**
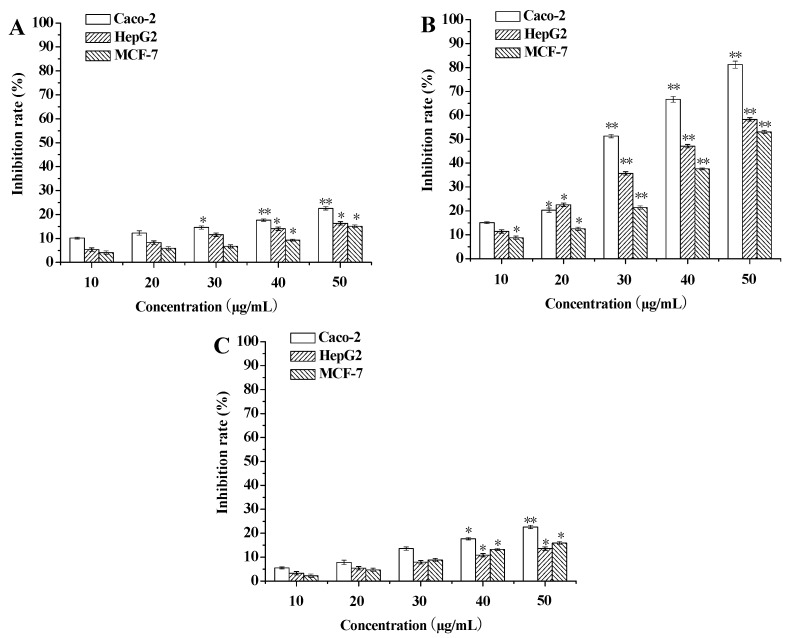
The cytotoxic effect of SBM extracts on three cancer cell lines. The cancer cells (Caco-2, HepG2 and MCF-7) were treated with various concentrations (0, 10, 20, 30, 40, 50 µg/mL) of GA (**A**), CGC (**B**) and EP (**C**) for 72 h, respectively. GA: gallic acid, CGC: cyanidin-3-*O*-glucoside chloride, EP: epicatechin. All results are the means ± standard deviation (SD) (*n* = 3). * *p* < 0.05 and ** *p* < 0.01, compared to the control group.

To evaluate cytotoxicity of SBM extracts against tumor cell lines, the MTT assay was used. The cytotoxic effects of GA, CGC and EP on Caco-2, HepG2 and MCF-7 cells were examined at different concentrations (Figure 3). The results indicated that CGC showed the significantly higher cytotoxic effects than GA and EP. In addition, three cancer cell lines (Caco-2, HepG2 and MCF-7) were more sensitive to the CGC than the others. CGC showed more than 50% cytotoxicity against three cell lines at 50 µg/mL. CGC-induced cytotoxicity on the three cancer cell lines was dose-dependent after a 72 h treatment. Caco-2 cells showed the highest susceptibility to the CGC with an IC_50_ value of 23.21 ± 0.14 µg/mL.

### 3.2. Changes of Cell Cycle Detected by Flow Cytometric Analysis

**Figure 4:**
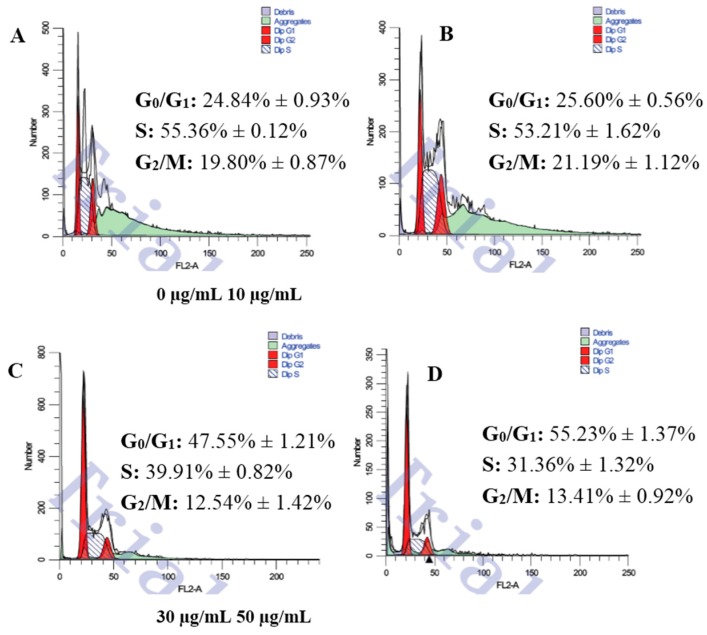
The effects of SBM on the cell cycle and the apoptosis rate of HepG2 cells. The cells (1 × 10^6^ cells/mL) were treated with 0 µg/mL (**A**), 10 µg/mL (**B**), 30 µg/mL (**C**) and 50 µg/mL (**D**) of CGC for 48 h, respectively. Cell cycle distribution and apoptosis were analyzed by flow cytometry. All data were expressed as the mean ± SD (*n* = 3).

To investigate the effect of SBM extract on the cell cycle distribution of Caco-2 cells, flow cytometry was used. As shown in Figure 4, the changes in the cell cycle progression of Caco-2 cells were notable. Compared to the CGC free group, as early as 4 h, the number of cells in the G_0_/G_1_ phase was significantly increased in a dose-dependent manner. Cell amount in the S and G_2_ phase did not present any trend. The progression of the cell cycle was arrested at the G_0_/G_1_ phase.

### 3.3. Flow Cytometric Analysis of Cell Apoptosis

**Figure 5:**
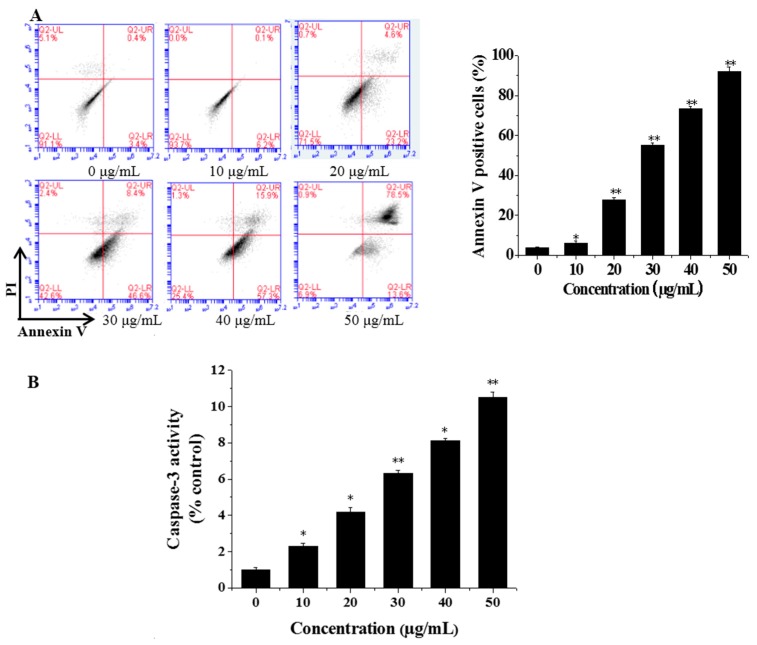
The analysis of Caco-2 cells apoptosis induced by CGC. (**A**) Flow cytometric analysis of MGC-803 cell apoptosis. Caco-2 cells were treated with CGC at 0 (control), 10, 20, 30, 40 and 50 μg/mL; (**B**) Caspase-3 activity in Caco-2 cells treated with 0, 10, 20, 30, 40 and 50 μg/mL of CGC, respectively. Caspase-3 activity was measured by caspase-3 colorimetric assay. All results are the means ± SD (*n* = 3). * *p* < 0.05 and ** *p* < 0.01, compared to control group.

Flow cytometry was used to identify and quantify the apoptosis and necrosis of the cells. Caco-2 cells were treated with CGC concentrations of 0, 10, 20, 30, 40 and 50 μg/mL. Then, cells were stained with Annexin V-FITC/PI and subsequently analyzed by flow cytometry. The four quadrants of the dual parameter fluorescent dot plots represented different states of the cells. The viable cells population was in the lower left quadrant (Annexin V−/PI−). The early apoptotic cells were in the lower right quadrant (Annexin V+/PI−) and the ones in late apoptosis were in the upper right quadrant (Annexin V+/PI+). As shown in Figure 5A, as early as 4 h, with the increasing concentration of CGC, the proportion of apoptotic cells increased. Both the Hoechst and flow cytometry results indicated that the CGC may induce apoptosis in Caco-2 cells.

### 3.4. CGC Activated Caspase-3

The caspase-3 activity in Caco-2 cells with treatment of CGC was measured by using a luminescent caspase activity assay kit (Thermo Fisher Scientific, Shanghai, China). As shown in Figure 5B, CGC increased caspase-3 activity in a dose-dependent manner, suggesting a possible relation between the CGC-induced apoptosis and the activation of caspase-3.

### 3.5. Effects of CGC on the mRNA and Protein Expression of Cell Cycle Protein (Cyclin D1)

**Figure 6:**
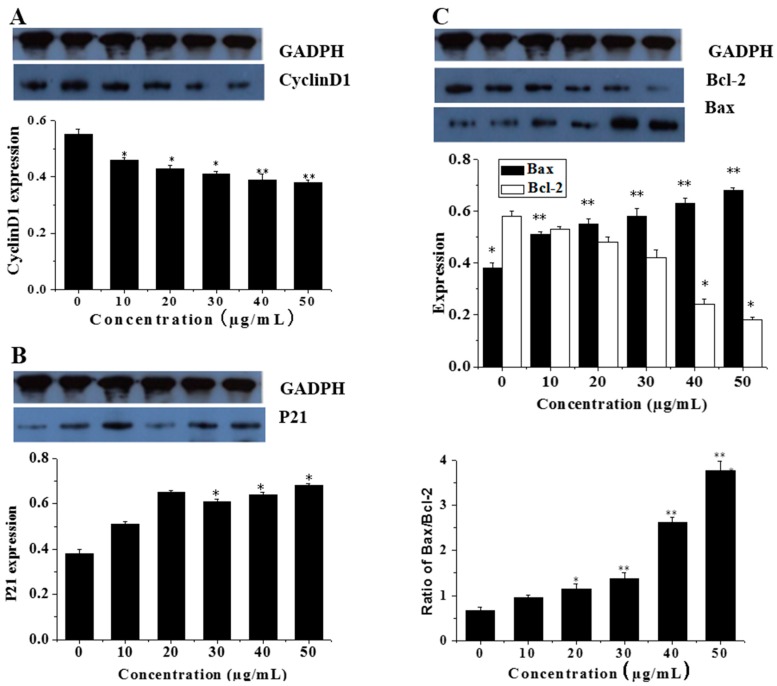
Western blotting analysis of apoptosis regulatory proteins in Caco-2 cells after treatment with increasing concentration of CGC. Densitometry was performed for the different proteins and normalized to the respective housekeeping control (**A**–**C**). *n* = 3, mean ± SD. * *p* < 0.05 and ** *p* < 0.01, compared to control group.

It was identified that CGC reduced the mRNA level of cyclin D1 (*p* < 0.001). Similarly, cyclin D1 protein level, detected by Western blotting, was downregulated (*p* < 0.001) (Figure 6A).

### 3.6. CGC Upregulated the Ratio of Bax/Bcl-2 and Downregulated the Expression of Mutant p21

To further investigate the effect of CGC on apoptosis regulatory proteins, we also examined the expression of p21, Bcl-2 and Bax in Caco-2 cells by immunocytochemistry analysis. As shown in Figure 6B,C, CGC significantly decreased the expression of Bcl-2 or p21 and increased the expression of Bax in a dose-dependent manner, which caused an increased ratio of Bax to Bcl-2. The results indicated the suppression of mutant p21, and the augment of the ratio of Bax/Bcl-2 might be the primary contributor to the apoptosis mediated by CGC.

## 4. Discussion

Polyphenols have been applied for treating cancer patients in traditional Chinese medicine [17]. The cytotoxicity of SBM was reported in our previous study [6], and the ethyl acetate fraction showed the strongest inhibition to cancer cell lines. In this paper, we further partitioned the ethyl acetate fraction and investigated the underlying mechanism. The cytotoxic effect of three polyphenols of SBM extract was screened in vitro against three cancer cell lines. CGC showed the most potent cytotoxicity on the colon cancer cell line Caco-2 with an IC_50_ value of 23.21 ± 0.14 μg/mL. Hence, CGC was scrutinized for the potential and mechanism against Caco-2 in vitro.

Moreover, it was found that anthocyanins (CGC) were more active than flavonoids (GA and EP). The cytotoxicity of anthocyanins has been well documented [18,19]. It has been shown that GA is quickly absorbed by Caco-2 cells and disappears after 24 h The disappearance may be due either to cell metabolism or oxidation [19,20]. Caco-2 cells are able to perform glucuronidation, sulfation, and methylation reactions, which provide a starting point for targeted identification of cell metabolites [21].

During the past decades, the killing of tumors through the induction of apoptosis has been recognized as a novel strategy for the identification of antitumor drugs [22]. Tumor cells have typically acquired damage to genes that directly regulate their cell cycles, and uncontrolled cell proliferation is the hallmark of cancer [23]. In cell culture, cell cycle arrest is a barrier to cancer, and senescence is a prerequisite for cancer cell cycle arrest [24]. Since cell proliferation was finely regulated by promoting or blocking cell cycle progression [3], the effect of CGC on cell cycle progression was examined. Figure 4 shows that CGC induced an accumulation of cells in S phase with an increasing apoptotic rate, which suggests that CGC induced apoptosis, perhaps through S phase arrest.

During apoptosis, DNA fragmentation and apoptotic body formation can be observed [3]. Cyclins are well-studied cell cycle regulators. Cyclin D1 is particularly required at the G_1_ to S phase [25]. Increased cyclin D1 expression has been shown in a number of primary human tumors and cell lines [26]. Over-expression of Cyclin D1 oncogenes has been shown to contract the G_1_ phase, decrease cell size and reduce the dependency of the cell on mitogens in animal models and cell lines [26]. In our study, CGC inhibited the cell proliferation via G_0_/G_1_ cell cycle arrest, accompanied with a decrease of Cyclin D1 both on the protein and mRNA level. Therefore, our results suggested that CGC suppressed Caco-2 proliferation by inducing cell cycle arrest and inhibiting Cyclin D1.

Apart from the cell cycle regulation, caspase family proteases play an important role in the mechanism of apoptosis [27]. Hence, we investigated whether caspase-3 was involved in CGC-induced apoptosis in Caco-2 cells, as it would contribute to the understanding of the mechanism responsible for apoptosis execution. Experimental results demonstrated that the higher the concentrations of CGC during treatment, the greater the expression of caspase-3 in Caco-2 cells (Figure 5B), which indicated that CGC could activate caspase-3 and caspase-3 might be involved in CGC-induced apoptosis.

It was reported that anti-apoptotic protein Bcl-2 and pro-apoptotic protein Bax are also important members involved in apoptosis [3]. Administration of p21 can have a substantial effect on tumor growth in vivo [28]. Furthermore, p21 treatment produced significant growth inhibition both in vivo and in vitro [28]. Some previous studies have demonstrated an inverse correlation between mutant p21 and Bax/Bcl-2 expression [29].

Based on this hypothesis, the expression of p21, Bcl-2 and Bax proteins in Caco-2 cells after the treatment with CGC was examined by immunocytochemical methods. In this study, immunocytochemical staining analysis showed that CGC enhances the expression of Bax and downregulates the expression of Bcl-2 and mutant p21 (Figure 6), which promotes the expression of caspase-3 to induce apoptosis. The results suggest that the mechanism of apoptosis may be associated with apoptosis-associated proteins taking part in intrinsic apoptosis pathways.

## 5. Conclusions

In conclusion, our results show that CGC exhibits cytotoxicity on Caco-2 cells in vitro and induces apoptosis in Caco-2 cells by activating caspase-3 and leading to S phase arrest, which might be related to upregulation of the ratio of Bax/Bcl-2 and downregulation of mutant p21. It is confirmed that SBM is effective against cancer cells in vitro, especially for colon cancer. Our study for SBM extract may discover that CGC might be used as a potential agent for colon cancer treatment.
